# Fast and Famous: Looking for the Fastest Speed at Which a Face Can be Recognized

**DOI:** 10.3389/fpsyg.2013.00100

**Published:** 2013-03-04

**Authors:** Gladys Barragan-Jason, Gabriel Besson, Mathieu Ceccaldi, Emmanuel J. Barbeau

**Affiliations:** ^1^Centre de Recherche Cerveau et Cognition, Université de Toulouse, CNRS-UMR 5549Toulouse, France; ^2^Institut de Neurosciences des Systèmes, INSERM UMR1106, Aix-Marseille UniversitéMarseille, France; ^3^Assistance Publique Hôpitaux de Marseille, Service de Neurologie et Neuropsychologie, Centre Hospitalier Universitaire La TimoneMarseille, France

**Keywords:** face recognition, familiarity, famous faces, go/no-go, SAB, speed constraints, memory

## Abstract

Face recognition is supposed to be fast. However, the actual speed at which faces can be recognized remains unknown. To address this issue, we report two experiments run with speed constraints. In both experiments, famous faces had to be recognized among unknown ones using a large set of stimuli to prevent pre-activation of features which would speed up recognition. In the first experiment (31 participants), recognition of famous faces was investigated using a rapid go/no-go task. In the second experiment, 101 participants performed a highly time constrained recognition task using the Speed and Accuracy Boosting procedure. Results indicate that the fastest speed at which a face can be recognized is around 360–390 ms. Such latencies are about 100 ms longer than the latencies recorded in similar tasks in which subjects have to detect faces among other stimuli. We discuss which model of activation of the visual ventral stream could account for such latencies. These latencies are not consistent with a purely feed-forward pass of activity throughout the visual ventral stream. An alternative is that face recognition relies on the core network underlying face processing identified in fMRI studies (OFA, FFA, and pSTS) and reentrant loops to refine face representation. However, the model of activation favored is that of an activation of the whole visual ventral stream up to anterior areas, such as the perirhinal cortex, combined with parallel and feed-back processes. Further studies are needed to assess which of these three models of activation can best account for face recognition.

## Introduction

The idea that face recognition is fast appears appealing (e.g., Bruce and Young, 1986; Jemel et al., 2010; Zheng et al., 2012). So what is the fastest speed at which a face can be recognized? Highly variable reaction times (RTs) have actually been reported in studies of face recognition, ranging from 400 to 900 ms (mean or median RTs, e.g., Kampf et al., 2002; Herzmann et al., 2004; Caharel et al., 2005; Anaki et al., 2007; Baird and Burton, 2008; Anaki and Bentin, 2009; Ramon et al., 2011; Barragan-Jason et al., 2012). Such variability can be accounted for by numerous factors, such as the number of stimuli (only one to hundreds), the use of repeated or trial-unique stimuli, and the nature of the stimuli (photographs or drawings). Moreover, the variety of protocols probably also accounts for some of this variability: yes/no (Caharel et al., 2007) but also priming (Lewis and Ellis, 2000) or category-verification (Rosch, 1975; Anaki and Bentin, 2009) tasks have been used. Intriguingly, whereas it is common to use challenging tasks in the field of object recognition, with the aim to address the fastest speed at which objects can be recognized (e.g., go/no-go task in Thorpe et al., 1996), this appears less common in the field of face recognition.

“Face recognition” is an ambiguous term however, as it can refer to either “top-down” or “bottom-up face recognition.” Top-down recognition corresponds to the situation of looking for someone in particular and involves the pre-activation of some diagnostic features about the person to be recognized (e.g., Lewis and Ellis, 2000; Tanaka, 2001). Such a paradigm can presumably be performed on the basis of a search for a few visual features and is expected to be fast. In contrast, bottom-up recognition corresponds to the situation of suddenly bumping into an acquaintance. This implies that subjects do not have any expectation about the face that will be presented, as in experiments where a large number of photographs with different identities are used. We can hypothesize that bottom-up recognition involves activation of representations in memory and thus would be rather slow. For example, in Lewis and Ellis (2000), different photographs of the same famous person (vs. another person) were presented and subjects had to press a different button for each identity. Authors reported that under such condition, face recognition was possible in 250 ms on average (after several repetitions) with an accuracy that could reach 100%. Even if this was not explicit, this task is clearly based on top-down face recognition processes. In contrast, subjects performed in an experiment by Kampf et al. (2002) a manual yes/no task on 144 famous faces presented among 244 unknown ones. Mean RT was 431 ms with an accuracy that varied between 67 and 91%. In this case, the task is based on bottom-up face recognition and RTs are much longer than in the previous study that relied on top-down face recognition. The fastest speed at which a face can be recognized thus clearly depends on the type of paradigm (top-down or bottom-up) used.

The speed at which a face can be recognized also depends on the strategy used by subjects (Bentin and Deouell, 2000). In particular, recognizing a familiar face in a bottom-up manner can be based either on familiarity only or on identification, familiarity being faster than identification (Yovel and Paller, 2004) in accordance with hierarchical models of face recognition (Bruce and Young, 1986). Furthermore, it has been hypothesized that participants may have difficulties preventing identification, that is, it is not enough to find a face familiar, one has to retrieve some knowledge about the identity of the person to behave appropriately (Bruce and Young, 1986; Valentine, 2001). Hence, most RTs reported to date may be rather long. This suggests that the speed of the fastest answers in face recognition tasks cannot be properly assessed if time constraints are not used to try to prevent participants from accessing the identification level.

Overall, no clear picture has emerged about the real speed of face recognition, mainly because no speed constraints have been used to our knowledge. However, the fastest RTs reported to date are around 400 ms. In Anaki et al. (2007), subjects had to categorize a large number of faces (180 famous and 180 unknown faces) using a yes/no paradigm. Mean RT was 411 ms. In a recent study by Ramon et al. (2011), minimum (not mean) RTs were around 370 ms in a task in which subjects had to recognize personally known faces (28 classmates) among unknown matched faces. It can thus be hypothesized that face familiarity should be at least as fast as 400 ms, and probably faster if speed constraints are used. Here, we report two experiments run with speed constraints and concentrate on bottom-up recognition. In the first experiment, participants had to recognize famous faces among unknown ones during a rapid go/no-go task. In the second experiment, we applied a new paradigm adding additional time constraints on responses.

## General Methods

### Experimental setting

Participants sat in a dimly lit room, 90 cm from a CRT computer screen. Stimuli were presented using Eprime 2.0 software and subtended a visual angle of ∼7.2 × 10.7. The photographs were displayed on a black background. To answer, participants had to raise their fingers from an infrared response pad as quickly as possible (e.g., Rousselet et al., 2003).

### Behavioral performance analyses

Performance (accuracy) and bias were computed using *d*′ and *C* measures (Snodgrass and Corwin, 1988). Based on the Signal Detection Theory, these measures reflect respectively the discrimination performance between targets and distractors and the strategy used (positive if conservative, negative if liberal). As 0 and 100% cannot be *Z*-transformed, a correction was applied where appropriate following Snodgrass and Corwin (1988). As some participants failed to do the task [χ^2^-test between hits and false alarms (FA), *p* < 0.05], their data were discarded from further analyses (see Table 1 for details). RTs <200 ms were considered as anticipation and were discarded from analyses (Rousselet et al., 2003; Barragan-Jason et al., 2012). To obtain an estimation of the minimal processing time required to recognize targets, the minimal behavioral reaction time (minRT) was computed by determining the latency at which correct go-responses (hits) started to significantly outnumber incorrect go-responses (FA; Rousselet et al., 2003; Bacon-Macé et al., 2007). For each condition, the analyses were performed either across trials (by pooling all trials from all participants for a given condition) and across participants (mean of all participants’ individual mean performances). Across-trial analyses have been used in previous studies (Rousselet et al., 2003; Barragan-Jason et al., 2012; Besson et al., 2012) and are like building a “meta-participant,” reflecting the performance over all the population. MinRTs across trials were computed using 10 ms time bins and determined as the middle of the first bin that was significant, χ^2^-test, *p* < 0.05, followed by at least three significant consecutive bins. Across participants, in order to allow for the lower statistical power than with across-trial data since there were fewer trials, we used 30 ms time bins and a Fisher’s exact test (*p* < 0.05), followed by at least two significant consecutive bins. Because of the non-normality of the data, Wilcoxon non-parametric tests (to compare bias of participants to 0) and non-parametric Spearman’s rank correlation coefficient were used.

**Table 1:** **Results for each experiment**.

		Experiment 1: speeded go/no-go	Experiment 2: SAB
Succeeded on the task/underwent the task	*N* (%)	31/31 (100%)	87/101 (86.1%)
**Accuracy (*d*′)**	Median	1.27	0.98
	First and third quartiles	[0.94; 1.51]	[0.59; 1.32]
	Range	[0.38; 1.82]	[0.33; 2.22]
	Across trials	1.11	0.90
**Bias (*C*)**	Median	0.55	0.07
	1st and 3rd quartiles	[0.34; 0.85]	[−0.13; 0.39]
	Range	[−0.13; 1.95]	[−1.00; 1.78]
	Across trials	0.54	0.09
Obtained a minRT	*N*	30/31 (96.8%)	57/87 (65.5%)
**Minimum RTs**	Median	555	420
	First and Third quartiles	[510; 600]	[390; 480]
	Range	[390; 780]	[360; 540]
Obtained a minRT at or before 420 ms	*N*	2/31 (6.5%)	32/87 (36.8%)
	Across trials	450	350

**M*, mean; *N*, number of participants; RT, reaction time; SD, standard deviation; minRT, minimal RT*.

## Experiment 1: Rapid Go/No-Go Categorization Task

### Participants

Thirty-one participants [17 female, median age: 24, (range: [19–37]), 27 right-handers] with normal or corrected-to-normal vision volunteered and gave their written informed consent to participate in the experiment.

### Stimuli

**Figure A1:**
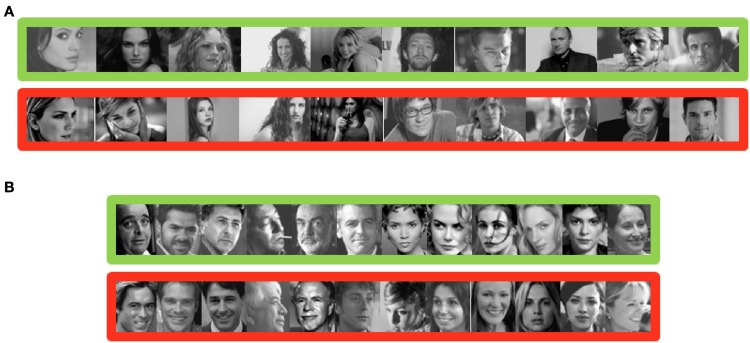
**Example of famous faces (green) and unknown faces (red) for experiment 1 in (A) and experiment 2 in (B)**.

Stimuli consisted of 540 photographs of unknown human faces and 270 photographs of famous faces (Brad Pitt, Bill Gates, etc.) chosen in a previous experiment as being recognized by people between 20 and 40 years old. All faces were presented in their natural context (i.e., they were not cropped and some background could be seen). Photographs of unknown faces were chosen to look like those of famous people in terms of quality (professional photographs), attractiveness (most of the photographs were of models) and emotion so that participants could not base their answers on these criteria. Each image was 320 × 480 pixels. Both series were comparable in luminance, contrast, number of pixels of the face in the image (manually cropped from their background). We also controlled other factors such as head orientation, emotion, and gaze (see supplementary Table 1 of Barragan-Jason et al., 2012). Examples of stimuli are provided in Figure A1A in Appendix.

### Protocol

**Figure 1:**
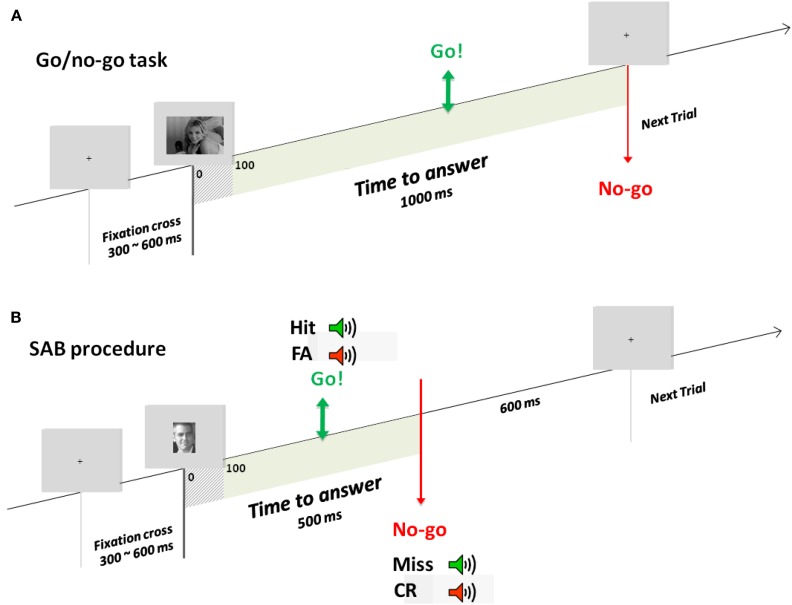
**Experimental designs**. Example of one trial in the go/no-go task **(A)** used in experiment 1 and the SAB procedure **(B)** used in experiment 2. During the go/no-go task, a fixation cross appeared for a random interval of 300–600 ms followed by the stimulus for 100 ms and a black screen which remained for 1000 ms. In the SAB procedure, a fixation cross appeared for a random interval of 300–600 ms followed by the stimulus presentation. A gray screen remained for 600 ms, which corresponded to the response deadline. If a go response was made before this response deadline, audio-feedback was played: positive if it was a target and negative if it was a distractor. In contrast, if a no-go response was made, positive audio-feedback was given at the response deadline if it was a distractor, negative if it was a target.

Experiment 1 consisted of a go/no-go recognition task (Famous/non-Famous recognition task) divided into three blocks of 180 (90 targets randomly chosen from the famous face face stimulus set and 90 distractors randomly chosen from the unknown face stimulus set. Random selection of images was done individually for each participant). Participants were trained before each condition with a specific set of stimuli. Participants were instructed to raise their fingers from an infrared response pad (go response) as quickly as possible when a target (a famous face) was presented. At the beginning of each trial, a fixation cross appeared for a random interval (300–600 ms), followed by a photograph flashed for 100 ms and a black screen for 1000 ms (Figure 1A). Stimuli were randomly displayed in blocks and among participants.

### Speed constraints

Stimuli were flashed quickly (100 ms) and participants had to answer before 1000 ms post-stimulus. Participants performed training sessions with dedicated stimuli before each task and they could repeat training if they wanted. After each block, including the training session, mean RTs, and false-alarm rates were displayed so that participants could monitor their performance. Lastly, they received strong encouragement before and between blocks to answer as fast as possible. In particular, after each block, they were asked to “beat” their RT score.

### Results

**Figure 2:**
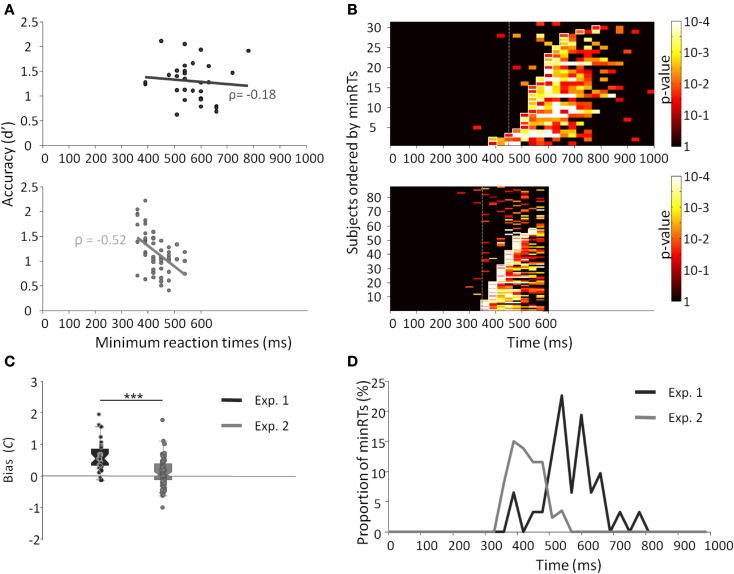
**Results from experiments 1 (dark gray) and 2 (light gray)**. **(A)** Minimal RTs for each participant according to *d*′ and their correlation. Each point represents a participant. **(B)** *p*-Value of the Fisher exact test computed between the number of hits and the number of false alarms within each bin of 30 ms. Lines represent individual participants, sorted by minRTs. **(C)** Boxplot of the bias (*C*) for both experiments. **(D)** Distribution of minimal RTs for both experiments.

**Figure 3:**
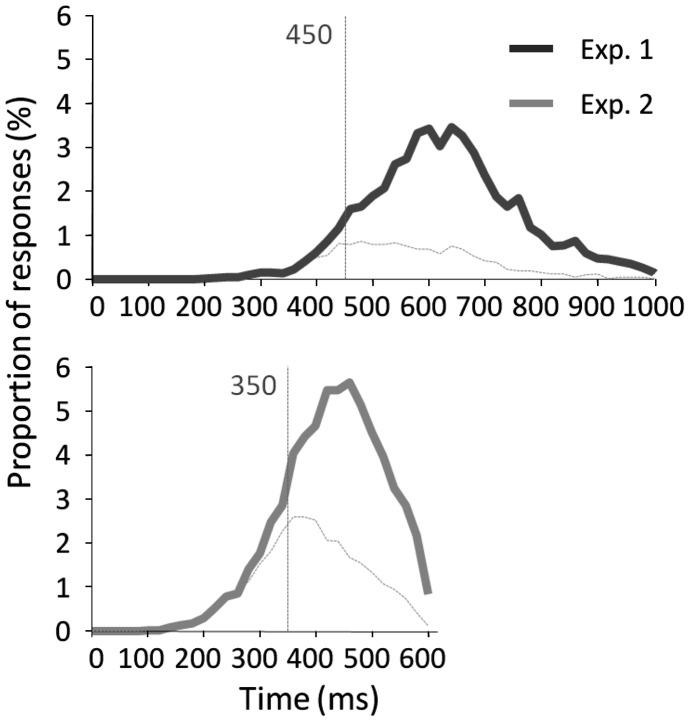
**RT distribution across trials with correct go-responses and false alarms for experiment 1 (top) and experiment 2 (bottom)**. Vertical lines indicate the minimal RT across trials.

Results across participants and across trials are presented in Table 1. Individual results are presented in Figures 2A,B. No participants failed to do the task and most of the participants performed it well (*d*′: median = 1.27). Participants used a rather conservative strategy, with a bias significantly different from 0 [Wilcoxon test, *Z*(31) = 488, *p* < 0.0001; *C*: median = 0.55; Figure 2C]. The median of minRTs across participants was 555 ms (range = [390–780]). Intriguingly, focusing on the fastest minRTs, 2 participants out of 31 (6.5%) had a minRT at 390 ms (with the following bin at 420 ms being empty, Figure 2D). No correlation was found between *d*′ values and minRTs (ρ = −0.18, *p* = 0.33; Figure 2A). Across trials, median RT was 626 ms and minimal RT was 450 ms. The RT distribution is presented in Figure 3.

### Discussion

This experiment is in agreement with a previous study (Barragan-Jason et al., 2012) and suggests that participants were rather slow overall since the median of minimal RTs was 555 ms, despite the use of speed constraints. However, the variability of RTs across participants was high. Intriguingly, two participants appeared much faster than the rest of the group, yet showed comparable accuracy. They appeared even faster than the across-trial minRT. This suggests that the speed constraints we used in this experiment may not have been optimal. We therefore tried to constrain participants to use an even faster strategy in Experiment 2.

## Experiment 2: The SAB Procedure: Extreme Speed Constraints

A recent study introduced the Speed and Accuracy Boosting (SAB) procedure (Besson et al., 2012). This novel protocol is assumed to constrain participants to use their fastest strategy and could thus allow assessing whether speed constraints in Experiment 1 were optimal or not. Basically, the SAB relies on a response deadline and audio-feedbacks to constrain subjects to answer both quickly and accurately (Figure 1B). We also increased the number of subjects (*N* = 101) in order to determine a robust minimal RT.

### Participants

A total of 101 participants (67 females) were included in this study (median age: 25, range: [18–30], all right-handers). All participants had normal or corrected-to-normal vision, volunteered, and gave their written informed consent to participate in the experiment.

### Stimuli

Stimuli consisted of 140 grayscale photographs of famous faces (corresponding to the images that were recognized with the best accuracy in experiment 1) and 140 unknown faces chosen randomly in the set of 540 unknown faces that remained the same for all participants. Because a previous study had shown that the SAB paradigm was highly demanding, all faces were cut out with the same size (208 × 279 pixels, visual angle: ∼4.7 × 6.3°) from their context. Stimuli were presented one by one, in the center of a gray screen. Examples of stimuli are provided in Figure A1B in Appendix.

### The SAB procedure

Based on a classical go/no-go task, the SAB constrains participants to answer before a response deadline following stimulus onset (Figure 1B). Here, based on earlier results (Besson et al., 2012), we used a response deadline of 600 ms (almost no subject can perform the task with a deadline at 500 ms). If a go response was made before this response deadline, an audio-feedback was played, positive if the item was a target (hit), negative if the item was a distractor (false-alarm). If a no-go response was made, audio-feedback was given at the response deadline, positive if the item was a distractor (correct no-go), or negative if the item was a target (omission). Before presentation of each item, a fixation cross was displayed for a pseudo-random time between 300 and 600 ms. Items were presented for 100 ms (included in the response deadline). Each experiment was preceded by two training blocks (for each training block: 10 target stimuli, to be recognized among 10 distractor stimuli). The task was made of one block but a self-paced pause was proposed every 20 items. A pause of minimum 30 s was provided in the middle of the experiment (after 140 items).

### Results

The results across participants and across trials are presented in Table 1. Individual results are presented in Figures 2A,B. Some participants (*N* = 14, i.e., 13.9%) failed to do the task, and their results were discarded. Participants used a slightly conservative strategy [*C*: median = 0.07; Wilcoxon test, *Z*(101) = 1403, *p* = 0.02; Figure 2C]. Median *d*′ for the other 87 participants was 0.98. A minRT could be calculated for 57 of the 87 participants. The median of minRTs across participants was 420 ms (range = [360–540]). A significant negative correlation was found between *d*′ values and minimal RTs (ρ = −0.52, *p* < 0.0001; Figure 2A). Across trials, median RT was 439 ms and minRT was 350 ms. RT distribution is presented in Figure 3.

Compared to Experiment 1, minRTs were clearly shifted to the left (i.e., proportionally more participants performed at optimal speed, Figure 2D). Additionally, a significantly different bias was observed between the two experiments, participants being more conservative in Experiment 1 than in Experiment 2 [*U*(31) = 2555, *p* < 0.0001; Figure 2C].

### Discussion

The main aim of this experiment was to determine the minimum RT needed to recognize a face. With a large population, this experiment showed that (1) a large proportion of participants showed individual minRTs faster than 400 ms; (2) 360 ms was the fastest individual minRT (350 ms across trials). Overall, this experiment brings strong evidence that face recognition is possible in ∼360 ms and probably not faster than this. However, and importantly, the fastest RTs were not much modified compared to Experiment 1.

## General Discussion

The present study investigates the minimal time strictly necessary to recognize a face. A large number of famous faces were used in both experiments, preventing subjects from relying on arbitrary or idiosyncratic visual clues (i.e., specific hair styles) that would have facilitated recognition. Our experiments thus clearly assess the speed of bottom-up recognition. In Experiment 1, we showed that participants could spontaneously recognize famous faces in as little as 390 ms during a challenging go/no-go categorization task (range: [390–780]). A large variability in RTs was observed among participants however. Fast participants (6.5%) could recognize faces in only 390 ms, whereas slow participants needed roughly 165 ms more to perform the same task. In Experiment 2, we therefore employed the SAB procedure, which adds a response deadline (here at 600 ms post-stimulus onset) to speed up responses and an audio-feedback (i.e., positive or negative) to optimize accuracy in order to encourage participants to use their fastest strategy. This highly demanding task was efficient since participants recognized famous faces faster, with a smaller time range [360–540 ms], and without any speed/accuracy trade-off. Across-trial minRTs shifted from 450 to 350 ms. However, the gain in individual fastest minRTs was relatively small as it shifted by only 30 ms, from 390 to 360 ms, suggesting that the fastest participants in Experiment 1 were already (and spontaneously) close to optimum performance. Across the two experiments, and despite different conditions, it thus appears that human participants can recognize famous faces in as little as 360–390 ms and that they cannot perform faster. This is much longer than the time needed to detect faces in natural scenes (250–290 ms, reviewed in Fabre-Thorpe, 2011) using similar behavioral paradigms. This lower behavioral bound at 360 ms is also an upper bound for neuronal processing. Given that about 100–130 ms are needed for decision and motor responses (Kalaska and Crammond, 1992; VanRullen and Thorpe, 2001b), this suggests that the fastest neural processes underlying face familiarity have occurred by 260 ms. This is clearly not in the time-window of the well-known component peaking at 170 ms in *M*/EEG studies and thought to index access to face representation. All in all, this suggests that face familiarity requires specific processes beyond face detection or access to face representation. These implications are discussed below.

Recognition memory has been largely described as being supported by two processes, familiarity and recollection (Mandler, 1980; Yonelinas and Levy, 2002; Yonelinas et al., 2010). The former (mere feeling that an item has been experienced previously) would be automatic and rapid, the latter (retrieval of specific contextual details) would be more effortful and time consuming (Juola et al., 1971; Brown and Aggleton, 2001; Rugg and Curran, 2007; Besson et al., 2012; Staresina et al., 2012). Therefore, we can hypothesize that a fast strategy relies merely on a feeling of familiarity, whereas a slow strategy involves the recollection of information about the person, which takes time. Most of the participants in Experiment 1 appeared to take some time to produce their response**s** compared to two “fast” participants. This appears to be in agreement with the idea that most are not satisfied by a mere feeling of familiarity but also need to identify the face (Bruce and Young, 1986; Valentine, 2001).

The SAB procedure was apparently successful at constraining participants to rely on a faster, familiarity-based, procedure since minRTs were clearly shifted toward a minimal boundary around 360–390 ms. Participants were found to be much less conservative in Experiment 2 than in Experiment 1 (Figure 2C), supporting the idea that participants used a different strategy. A conservative strategy would be expected if participants were allowed some time (even under speed constraints but without a response deadline) so that they could optimize the hit rate and diminish FA, which are socially inadequate. However, because stimuli were different between experiment 1 and 2, it is possible that just using stimuli of experiment 2 in experiment 1 would have generated the results observed with the SAB procedure. It could be interesting to verify this point in another study. Overall, both studies converge to a minimal boundary at 360–390 ms.

What can this 360–390 ms time limit tell us about underlying neural mechanisms? Considering the literature on rapid object categorization, it seems that about 250–290 ms are needed to produce reliable behavioral respons**es** when categorizing visual objects (Fabre-Thorpe et al., 2001; VanRullen and Thorpe, 2001a; Rousselet et al., 2003; Joubert et al., 2007; Macé et al., 2009; Fabre-Thorpe, 2011). Knowing that the time to trigger a manual response is around 100–130 ms (Kalaska and Crammond, 1992; VanRullen and Thorpe, 2001b), object categorization would rely on neural activity starting at about 120 ms. Within this framework, Dicarlo et al. (2012) suggest that object recognition is consistent with feed-forward inter-area processing involving about 10 synapses from the retina to high level visual areas [infero-temporal cortex (IT)]. Considering that 10 ms are needed to transfer information between each of these different areas, Dicarlo et al. (2012) proposed that a first high level representation of the object can be available around 100 ms after its presentation (e.g., Desimone et al., 1984; Logothetis and Sheinberg, 1996; Liu et al., 2009). This feed-forward hypothesis is consistent with recent behavioral studies showing that participants can initiate saccades toward visual objects (faces, animals) in as little as 100–120 ms after stimulus onset (Kirchner and Thorpe, 2006; Crouzet et al., 2010).

Here, we find that recognizing faces takes ∼100 ms longer than object categorization. It could be proposed that face recognition relies on the same feed-forward mechanisms that have been posited for object categorization but with the recruitment of additional areas higher up in the visual stream. Visual recognition has traditionally been considered as a hierarchical process where visual representations become gradually more invariant and specific along the pathway. Familiarity with a face requiring the highest level of specificity, visual areas in the highest area of the visual ventral pathway, such as the perirhinal cortex or even the temporal pole, could be recruited for such a process. Evidence in favor of the implication of the anterior temporal lobes in person processing are numerous, including studies of brain-lesioned patients suffering from person agnosia (Joubert et al., 2004, 2006) and fMRI studies (e.g., Haxby et al., 2000). Furthermore, medial temporal lobe structures such as perirhinal and entorhinal cortex or hippocampus could be involved in these processes, as their role in recognition memory tasks has often been underlined (Bowles et al., 2007; Diana et al., 2007; Montaldi and Mayes, 2010; Staresina et al., 2012) and as it is also in these areas that “person-specific” neurons are found (Quiroga et al., 2005). The perirhinal cortex, in particular, is the highest area in the visual pathway and is thought to be a critical node for familiarity (Barbeau et al., 2005, 2011; Aggleton and Brown, 2006).

If face recognition is only feed-forward, roughly 10 additional synapses should be activated. However, direct projections have been reported from IT to the perirhinal and temporal pole (Suzuki and Amaral, 1994a,b) so that the additional time necessary should be 20–30 ms at the very most. Thus, it appears implausible that face recognition should rely on a purely feed-forward process.

Another possibility, proposed by Hochstein and Ahissar (2002), distinguishes vision-at-a-glance based on feed-forward activity, from vision-with-scrutiny, based on processes beginning at the top of the hierarchy and “gradually returning as needed” to the ventral stream (Hochstein and Ahissar, 2002; Hegdé, 2008) in order to refine perceptual representations (Bullier et al., 2001). In line with this idea, an early activity in response to faces was observed at 130 ms in the anterior frontal gyrus, which could signal to areas at the top of the hierarchy that a face has been detected (Bar et al., 2006; Barbeau et al., 2008). Furthermore, a period of massively parallel processing has been identified in the whole visual ventral stream at 240 ms during a famous/unknown recognition task in intracerebral recordings in epileptic patients (Barbeau et al., 2008). Additionally, such latency could correspond to a differential activity between famous and unknown faces in the perirhinal cortex (Trautner et al., 2004). It is noteworthy that latencies in the hippocampus are delayed by about 80–100 ms compared to those of the perirhinal cortex (Trautner et al., 2004; Barbeau et al., 2008; Mormann et al., 2008). Considering the 100–130 ms necessary to trigger a manual response, this neural activity at 240 ms could be the basis for the first reliable behavioral responses at 360 ms observed in this study. Hence, face recognition would not rely on a first pass of feed-forward activity, but would more plausibly involve parallel processes implying both local and inter-area feed-back communication within and among the whole ventral stream.

An alternative can be formulated, however. A core network for face processing involving the Occipital Face Area (OFA), the Fusiform Face Area (FFA), and the posterior superior temporal sulcus has been identified and studied extensively (Haxby et al., 2000, 2001; Gobbini and Haxby, 2006). Because these areas, the FFA and OFA in particular, show an adaptation effect for identity, it has been proposed that this core network could be involved in representing individual faces (Rossion, 2008, 2009). Consequently, this core network could be sufficient to support face recognition. Furthermore, based on the study of patients with brain lesions, it has been proposed that this core network does not rely on feed-forward processing (from posterior occipital areas to the OFA to the FFA) but on connections from posterior areas to the FFA, then back to the OFA using reentrant loops (Rossion, 2008, 2009). Hence, the latency we observe for familiarity-based recognition could be due to the time needed for this reentrant processing to take place, for instance to refine visual information. We believe this is a reasonable alternative to the idea put forward above that face familiarity would require the whole visual ventral stream and, to our mind, this alternative merits further investigation. At this stage however, we note that the activity of this core network is probably reflected in the component peaking around 170 ms recorded using *M*/EEG. This component is supposed to reflect configural/holistic processes (e.g., Gosling and Eimer, 2011) but some studies suggest that it may also reflect individuation/recognition processes (e.g., Jemel et al., 2010). Behavioral latencies reported in the current study appear incompatible with this early component. Furthermore, such a scheme would discard the role of the perirhinal cortex in face recognition whereas, as already mentioned, this area appears to be a key node in familiarity, and faces elicit prominent activity in this region (Trautner et al., 2004; Dietl et al., 2005; Barbeau et al., 2008).

It should be noted that a potential limit to our study is that stimuli were different in Experiments 1 and 2, implying that performance of the two tasks cannot be directly compared. However, this is not a major problem since results in Experiments 1 and 2 are actually congruent on the idea that the fastest individual RTs occur around 360–390 ms, which is the main emphasis of this study. Although more subjects performed around 360–390 ms in Experiment 2, the use of easier stimuli and stronger speed constraints did not allow them to be much faster than in Experiment 1. Additionally, previous studies that used different paradigms or type of stimuli reported behavioral latencies that seem consistent with our results (418 ms in Anaki et al., 2007; 380 ms in Ramon et al., 2011; 431 ms in Kampf et al., 2002). Anaki et al. (2007) and Kampf et al. (2002) studies were quite similar to our experiments: they used similar categories of faces (famous vs. unknown) and a comparable number of stimuli (180 targets vs. 180 distractors in Anaki et al. and 144 vs. 288 in Kampf et al.), i.e., subjects were in a bottom-up recognition situation. However, these authors used yes/no tasks rather than a go/no-go task as here; they didn’t use any speed constraints and they focused on mean rather than minimal RTs. In contrast, Ramon et al. (2011) reported minimum RTs but a small set of faces was used (28 faces of classmates). The impact of this restricted set of faces on speed remains to be determined. These previous studies suggest that the lower bound for the fastest RTs were reached independently of the conditions and stimuli used, confirming our current results performed under speed constraints and in a bottom-up situation.

In conclusion, the current study has determined that the very minimal RT to recognize famous faces among unknown ones is around 360–390 ms after stimulus onset. It seems that such latencies are consistent with the speed of recognition memory in general (i.e., objects and abstract patterns, Besson et al., 2012), raising the question of whether the familiarity processes used to recognize faces are face-specific or are related to a general familiarity system. The SAB procedure seems an efficient method to determine the minimal time needed to perform a cognitive task. Such a method could be used to study other kinds of familiar faces, such as personally known faces (Ramon et al., 2011) or newly learned faces (Tanaka et al., 2006). We have discussed which model of activation of the visual ventral stream could account for such latencies. Three models were proposed and the one favored to date is that of an activation of the whole visual ventral stream up to anterior areas such as the perirhinal cortex, combined with parallel and feed-back processes. Further studies are needed to assess these three models of activation better.

## Conflict of Interest Statement

The authors declare that the research was conducted in the absence of any commercial or financial relationships that could be construed as a potential conflict of interest.
